# Electromagnetic Exposure Assessment of 5G Mobile Phones: SAR and Thermal Distribution in a Multi-Layer Human Head Model

**DOI:** 10.3390/s26051468

**Published:** 2026-02-26

**Authors:** Dengpeng Chen, Bingtao Zhang

**Affiliations:** 1 School of Electronic Information, Xi’an Railway Vocational & Technical Institute, Xi’an 710014, China; 08011071@xatzy.com; 2 School of Electronics and Information Engineering, Lanzhou Jiaotong University, Lanzhou 730070, China

**Keywords:** 5G mobile communication, electromagnetic exposure assessment, multi-layer human head model, numerical simulation, SAR, temperature distribution

## Abstract

The rapid deployment of 5G technology has raised public concern regarding the potential health effects of electromagnetic radiation from mobile devices. This study systematically evaluates the specific absorption rate (SAR) and temperature distribution in a multi-layer spherical head model exposed to near-field radiation from a 5G mobile phone antenna. A planar inverted-F antenna (PIFA) covering the 3.5 GHz band was integrated into a smartphone model, and simulations were performed in COMSOL Multiphysics 6.3 under input powers of 21 dBm and 24 dBm at varying antenna–head distances. The results show that the peak SAR in the brain layer remained at 0.034 W/kg and 0.065 W/kg for the two power levels, both well below the International Commission on Non-Ionizing Radiation Protection (ICNIRP) safety limit of 2 W/kg. The highest SAR occurred in the scalp layer, decreasing gradually through the skull and brain tissues. After 30 min of exposure, the maximum brain temperature reached only 37.223 °C, far lower than the thermal damage threshold. Increasing the antenna–head distance from 5 mm to 30 mm reduced SAR by up to 50.2%, while temperature variations remained negligible (≤0.18%). These findings demonstrate that under typical usage conditions, 5G mobile phone radiation complies with international safety standards and poses no significant thermal risk, thereby contributing to a deeper understanding of bio-electromagnetic interactions and supporting ongoing wireless-communication safety assessments.

## 1. Introduction

The rapid development of 5G technology is driving the upgrading of smartphones [1,2], and whether the electromagnetic radiation of mobile phones has an impact on human health has been a hot topic of widespread concern for nearly 20 years. The Institute of Electrical and Electronics Engineers (IEEE) and the International Commission on Non-Ionizing Radiation Protection (ICNIRP) have established relevant limits for human electromagnetic field exposure [3,4,5].

When the human head is exposed to electromagnetic radiation from a mobile phone for an extended period, biological tissues absorb the electromagnetic radiation energy. Research on the absorption of electromagnetic waves by biological tissues began decades ago [6]; Bhargavaa et al. [7], used the Finite-Difference Time-Domain (FDTD) algorithm to study the effect of a 900 MHz rectangular microstrip antenna on the SAR and temperature rise of the human head under different mobile phone usage methods. Michelant L et al.’s [8] study provides the first controlled human experimental evidence that localized exposure to 26 GHz millimeter-wave radiation, at power densities compliant with international safety guidelines, does not significantly modulate resting-state electroencephalographic (EEG) activity in adult volunteers. Miura et al. [9] studied the superposition of temperature rise when the human body is simultaneously exposed to a 2 GHz inverted F antenna and a 28 GHz patch antenna array, and evaluated the temperature rise levels under different SAR values and absorption power density ratios. Xue Yizhe et al. [10] studied the effects of radiofrequency radiation from 5G mobile phones on sperm quality and sex hormone levels in adult male mice.

In near-field radiation, the absorption of electromagnetic radiation by biological tissues depends on multiple factors, such as the structure of the mobile phone antenna, its operating frequency, input power, radiation time, and the distance between the antenna and the human head [11,12,13,14], Therefore, it is necessary to systematically study the impact of mobile phones on the specific absorptivity (SAR) of the human head under common operating frequencies, different antenna input powers, and variations in the distance between the human head and the antenna, in order to fully understand the biological effects of mobile phone electromagnetic radiation on the human head.

To more realistically simulate the exposure of the human head to the electromagnetic radiation environment of a 5G mobile phone, this paper designs an antenna model that can cover common operating frequency bands of 5G mobile phones based on the Nashaat DM [15] planar inverted-F antenna (PIFA) by tuning and optimizing the antenna structure. Then, a 5G mobile phone model and a three-layer spherical head model representing the skin layer, skull layer, and brain layer of the human head are built in COMSOL, Multiphysics 6.3. Scattering boundary conditions are used to truncate the head model from the external free space, thereby simulating that the human head is in the entire free space. Using Maxwell’s equations, a comparative analysis is conducted to provide a theoretical basis for whether the electromagnetic radiation of the mobile phone affects the SAR distribution of the human head under actual conditions with an input power of 21 dBm and 24 dBm [16], and operating at the most typical 5G frequency [17], currently planned globally (operating frequency: 3500 MHz). At the same time, the effects of changes in radiation time and distance on the SAR values of the human scalp, skull layer, and brain layer when the 5G mobile phone is operating at 3.5 GHz are also studied.

Despite numerous studies on electromagnetic exposure, most prior works focus on frequencies below 3 GHz or use simplified anatomical models. After condensing, the main contributions of this study can be summarized as follows: (1) evaluating a multi-layer spherical head model under typical 5G frequency (3.5 GHz), (2) integrating a realistic PIFA covering multiple 5G bands, (3) systematically analyzing both SAR and thermal distributions under varying input powers and antenna–head distances, and (4) providing a comparative assessment of distance-dependent exposure reduction. Our work thus offers a timely and realistic assessment of 5G mobile phone exposure under typical usage scenarios.

This study is organized as follows: Section 1 (Introduction) has been organized to first present the technological background and public health concerns regarding 5G mobile-phone radiation, review existing safety standards (e.g., ICNIRP, IEEE) and prior research on specific absorption rate (SAR) and thermal effects in biological tissues, and finally identify the research gap and state the objectives of this study. Section 2 describes the human head model, antenna design, and numerical methods. Section 3 presents the simulation results, including SAR and temperature distributions, and discusses the effects of distance and exposure time. Section 4 concludes this study and suggests future research directions.

## 2. Model Establishment and Method Research

### 2.1. Human Head and Mobile Phone Antenna Model

**Figure 1:**
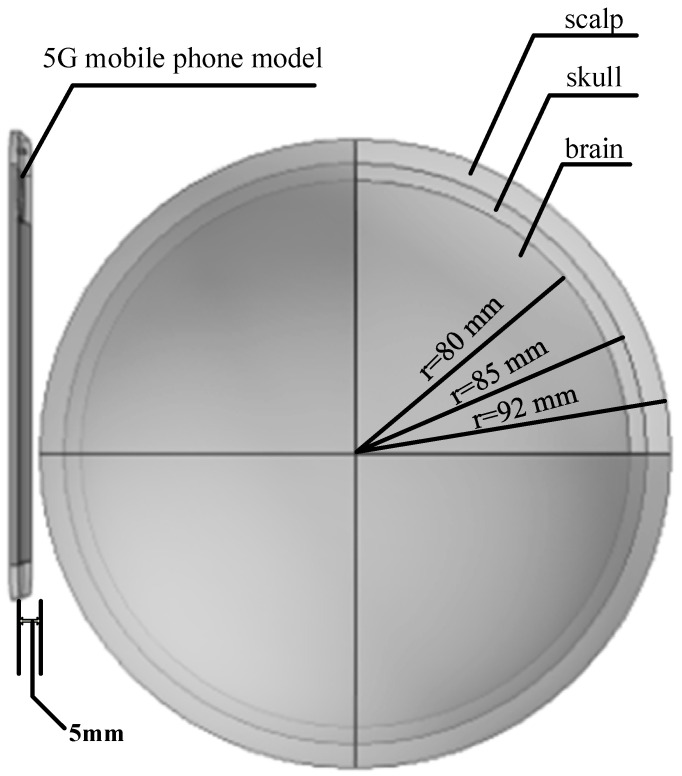
Human head and 5G mobile phone model.

**Figure 2:**
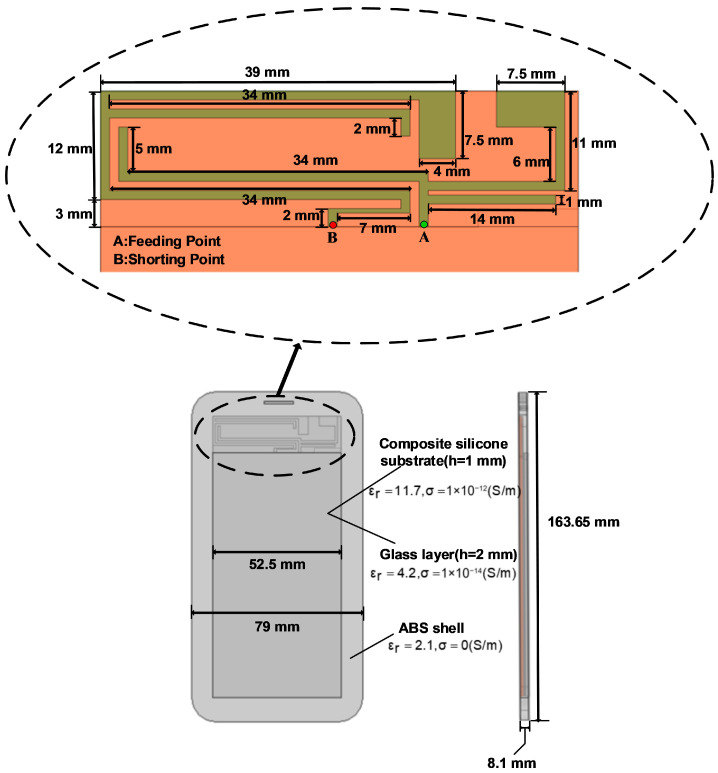
5G mobile phone antenna model.

Exposing the human head directly to electromagnetic radiation for experimental measurements is considered, to some extent, unethical [6,7,8], Therefore, it is more reasonable to simulate the human head tissue under the electromagnetic radiation environment of a mobile phone as realistically as possible by building a numerical model [16,17], The human head model [18], and 5G mobile phone model used in this study are shown in Figure 1. The human head model consists of three layers: skin layer, skull layer, and brain layer, with radii of 92 mm, 85 mm, and 80 mm, respectively. The 5G phone model is similar in size to the Huawei Mate 60. Its main components include a radiating patch antenna (52.5 × 15 mm^2^), a dielectric substrate (52.5 × 115 × 0.8 mm^3^), a composite silicone substrate (52.5 × 100 × 1 mm^3^), a glass layer (52.5 × 100 × 2 mm^3^), and an ABS shell (79 × 163.65 × 8.1 mm^3^). Detailed parameters are shown in Figure 2.

The canonical PIFA model used in this study does not incorporate a dedicated backside reflector. A reflector, typically a ground plane, placed between the radiating element and the user’s head is a well-established method to reduce the Specific Absorption Rate (SAR) by decoupling the antenna and directing the electromagnetic energy away from the user. However, this improvement in exposure safety comes with a trade-off: the radiation pattern becomes more directional. While beneficial in fixed scenarios, a directional pattern may compromise the robustness of the communication link in real-world mobile usage, where the orientation of the handset relative to the base station is constantly changing. The near-omnidirectional pattern of our modeled antenna, though resulting in a comparatively higher simulated SAR near the head, represents a common configuration in many consumer smartphones that prioritize consistent connectivity. This baseline analysis is crucial for understanding the potential exposure reduction benefits that could be achieved through more advanced, exposure-aware antenna designs without unduly degrading the quality of service.

The exposure assessment presented thus far is based on a single-element, near-omnidirectional antenna. To address the evolution of mobile technology, it is pertinent to discuss the implications of advanced antenna systems like beam forming and Multiple-Input Multiple-Output (MIMO), which are foundational to 5G and beyond. These technologies enhance capacity and coverage by dynamically shaping radiation patterns and multiplexing signals in space. However, the intelligence and adaptability inherent in these systems also introduce a powerful new paradigm for proactive exposure management. Unlike static antennas, smart antenna arrays can leverage real-time channel state information, user positioning data, or predefined safety protocols to implement “spatial avoidance” strategies.

As can be seen in Figure 1, the 5G phone antenna model is located 5 mm to the left of the head model, serving as a near-field radiation source. The input power of the phone antenna is set to 21 dBm and 24 dBm respectively, Since the different tissue layers of the human head have different material properties at different frequencies, a fourth-order Cole–Cole model [19], is adopted, as shown in Formula (1). At the same time, it is assumed that the medium of each tissue layer is uniform, so the relative permittivity and conductivity of each tissue layer of the human head are approximately calculated. As shown in Table 1.

(1) $${\hat{\varepsilon }}_{r}={\varepsilon^{\prime }}_{r}-j{\varepsilon^{\prime \prime }}_{r}=\varepsilon_{r\infty }+\sum_{n=0}^{4}\frac{\Delta \varepsilon_{rn}}{1+{\left(j\omega \tau_{n}\right)}^{1-\alpha }}+j\frac{\sigma_{i}}{\omega \varepsilon_{0}}$$

where: ${\hat{\varepsilon }}_{r}$ is the complex relative permittivity; $\varepsilon_{r}^{\prime }$ is the relative permittivity (i.e., the real part of ${\hat{\varepsilon }}_{r}$); $\varepsilon_{r}^{\prime \prime }$ is the loss factor (i.e., the imaginary part of ${\hat{\varepsilon }}_{r}$); ${\hat{\varepsilon }}_{r}$ is the relative permittivity at the optical frequency; $\Delta \varepsilon_{rn}$ is the increment of the relative permittivity; $\tau_{n}$ is the center relaxation time; α is the relaxation distribution time, taking the value of 0≤α≤1; $\sigma_{i}$ is the ionic conductivity; ω is the angular frequency.

Table 1 shows the dielectric properties of the scalp, skull, and brain tissue layers in the human head model used in this study at 3500 MHz, including the relative permittivity ($\varepsilon_{r}$) and conductivity (σ).

### 2.2. Electromagnetic Wave Propagation Analysis in the Human Head Model

#### 2.2.1. Analysis of Electromagnetic Wave Propagation Equations

Considering that mobile phone electromagnetic waves propagate in free space, the intensity of electromagnetic waves decreases with increasing incident distance due to heat loss. Therefore, based on the above complex factors, in order to simplify the analysis and calculation, the following assumptions were made in this study:

1. Simulate free space propagation of electromagnetic waves using an air domain with a wavelength greater than 1/4 λ [20].

2. Electromagnetic waves directly contact the human head in the air domain and are completely absorbed by the human head tissue [7,8].

3. Use scattering boundary conditions to truncate the free space outside the head model [7,8,9,10].

4. The dielectric properties of the tissue are uniform and constant [7,8,9,10].

5. For the purpose of this study, a continuous-wave (CW) excitation at the carrier frequency of 3.5 GHz is assumed.

The antenna of the 5G mobile phone model located on the left side of the head model is fed by a lumped port and emits electromagnetic waves into the air domain, The electromagnetic waves penetrate the three-layer head model, and each tissue layer absorbs its electromagnetic radiation. Usually, Maxwell’s equations are used for numerical solution. However, in order to simplify the calculation, the model solves the vector Helmholtz equation of each tissue layer within a specific frequency, as shown in Formula (2) [7,8,9,10].

(2) $$\nabla \times \frac{1}{\mu_{r}}\nabla \times E-{k_{0}}^{2}\varepsilon_{r}E=0$$

where E represents the electric field strength (V/m), $\mu_{r}$ is the relative permeability, $\varepsilon_{r}$ is the relative permittivity, $k_{0}$ is the free space wave vector (m^−1^).

#### 2.2.2. Assumptions of Electromagnetic Wave Propagation Boundary Conditions

**Figure 3:**
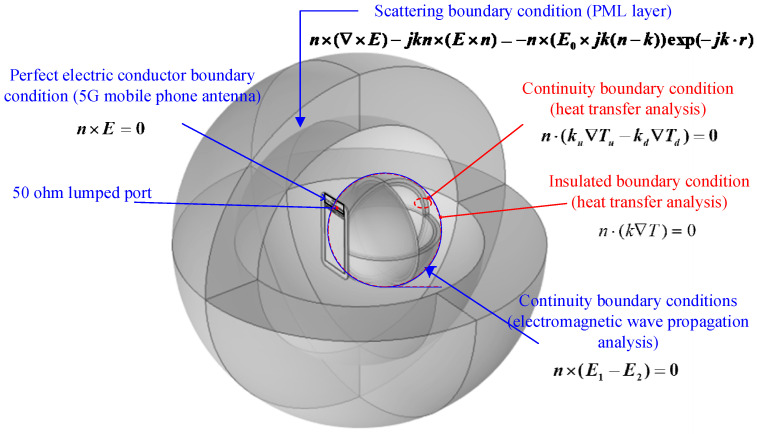
Boundary conditions for analysis of electromagnetic wave propagation and heat transfer.

The radiating antenna located at the top of the 5G mobile phone emits electromagnetic waves into the surrounding space and propagates into free space with a specific radiated power, The human head model absorbs its electromagnetic radiation energy. The radiating patch antenna is fed by a lumped port. As shown in Figure 2, point A is the 50 ohm characteristic impedance feeding point, and point B is the short-circuit point, thus generating an electromagnetic field around the radiating antenna. The boundary condition analysis of electromagnetic wave propagation is shown in Figure 3. The boundary conditions between different dielectric layers are assumed continuous to model transmission, while scattering boundary conditions and a PML domain are used to simulate free-space propagation and minimize reflections. The specific description is as follows:

The radiating patch antenna is defined as the boundary condition of an ideal conductor, expressed by Formula (3) [21].

(3) n×E=0

The boundary conditions between different dielectric layers in the model, such as the boundary condition between the air domain and the scalp layer, are assumed to be continuous, expressed by Formula (4) [7,8,9,10].

(4) $$n\times (E_{1}-E_{1})=0$$

In order to avoid the reflection of the emitted electromagnetic waves, a 3D spherical perfectly matched layer (PML) domain is added around the air domain of the human head model. At the same time, the outer surface of the PML, domain is set as the scattering boundary condition. It is expressed by Formula (5) [7,8,9,10].

(5) $$n\times (\nabla \times E)-jk^{\prime }n\times (E\times n)=-n\times (E_{0}\times jk^{\prime }(n-k^{\prime }))\exp(-jk^{\prime }\cdot r)$$

where $k^{\prime }$ is the free space wave vector (m^−1^), σ is conductivity (S/m), n is the normal vector, and $j=\sqrt{-1}$, $E_{0}$ represents the incident plane wave (V/m).

#### 2.2.3. SAR Value Analysis

The 5G mobile phone radiating patch antenna radiates electromagnetic waves in free space. The electromagnetic waves interact with the human head tissue, causing the electromagnetic wave radiation energy to be absorbed by different tissue layers in the human head model. The absorbed electromagnetic wave energy is measured using the specific absorptivity SAR [22], as shown in Formula (6).

(6) $$SAR=\frac{\sigma }{2\rho }{\left|E\right|}^{2}$$

where E represents the electric field strength (V/m), σ is conductivity (S/m), and ρ is the tissue density ${(\mathrm{kg}/m}^{3})$.

### 2.3. Heat Transfer Analysis in Human Head Model

The human head model is exposed to the 3D space of the electromagnetic radiation of the 5G mobile phone antenna. In order to study the temperature field distribution of various layers of human head tissues, according to Maxwell’s equation, the heat loss generated by the absorption of electromagnetic radiation by biological tissues, and the heat conduction between tissues and blood, are established. The Pennes bioheat equation [6,7,8,9], which is composed of three parts of metabolic heat source, is used to get the temperature field distribution of each tissue layer of the human head model. Table 2 shows the thermal properties of biological tissues in the scalp, skull, and brain layers of the human head model.

In order to simplify the study of the problem, the following assumptions were proposed when establishing a three-dimensional biological heat transfer model:

1. The three-layer biological tissue of the human head model has uniform and constant thermal properties [7,8,9,10].

2. There is no energy exchange problem in the human head model [7,8,9,10].

3. No chemical reaction in each tissue layer [7,8,9,10].

4. In the initial stage, the temperature distribution in the human head is uniform at 37 °C.

#### 2.3.1. Analysis of Heat Transfer Equation

The Penns transient biological heat equation [23,24,25] is used to describe the occurrence of heat transfer, and the temperature field distribution of each layer of the human head is analyzed. The heat transfer equation is expressed by Formula (7).

(7) $$\rho C\frac{\partial T}{\partial t}=\nabla \cdot (k\nabla T)+\rho_{b}C_{b}\omega_{b}(T_{b}-T)+Q_{met}+Q_{ext}$$

where ρ is the tissue density ${(\mathrm{kg}/m}^{3})$, C is the tissue specific heat capacity (J/kg·°C), k is the thermal conductivity (w/m·°C), T is the tissue temperature (°C), $T_{b}$ is the blood temperature (°C), $\rho_{b}$ is the blood density ${(\mathrm{kg}/m}^{3})$, $C_{b}$ is the blood specific heat capacity (J/kg·°C), $\omega_{b}$ is the blood irrigation rate (1/s), $Q_{met}$ is the metabolic heat source ${(w/m}^{3})$, and $Q_{ext}$ is the external heat source ${(w/m}^{3})$.

The heat conduction between tissue and blood flow is approximately $\rho_{b}C_{b}\omega_{b}(T_{b}-T)$. The external heat source is the heat loss caused by the absorption of electromagnetic radiation by biological tissues. It can be defined as Formula (8).

(8) $$Q_{ext}=\frac{1}{2}\sigma_{tissue}{\left|E\right|}^{2}=\rho \cdot SAR$$

where $\sigma_{tissue}=2\pi f\varepsilon_{r}^{\prime }\varepsilon_{0}$ is the relative dielectric constant of tissue and $\varepsilon_{0}$ is the (absolute) dielectric constant in vacuum.

#### 2.3.2. Heat Transfer Boundary Condition Assumptions

When conducting heat transfer analysis, we assume no heat flux across the outer boundary, thereby focusing solely on the internal heat transfer mechanisms between tissue layers. Thermal scattering of tissues into the surrounding space. Assumed that the surface of the scalp layer is a thermal boundary condition is expressed by the Formula (9) [6,7,8,9].

(9) n⋅(k∇T)=0

It is also assumed that there is no contact resistance between the scalp and skull layers and the skull and brain layers in the human head model. That is, the boundaries of the tissue layers in the human head model are continuous. The continuous boundary condition is defined as shown in Formula (10) [6,7,8,9].

(10) $$n\cdot (k_{u}\nabla T_{u}-k_{d}\nabla T_{d})=0$$

## 3. Results and Discussion

### 3.1. Model Validation and Benchmarking

**Figure 4:**
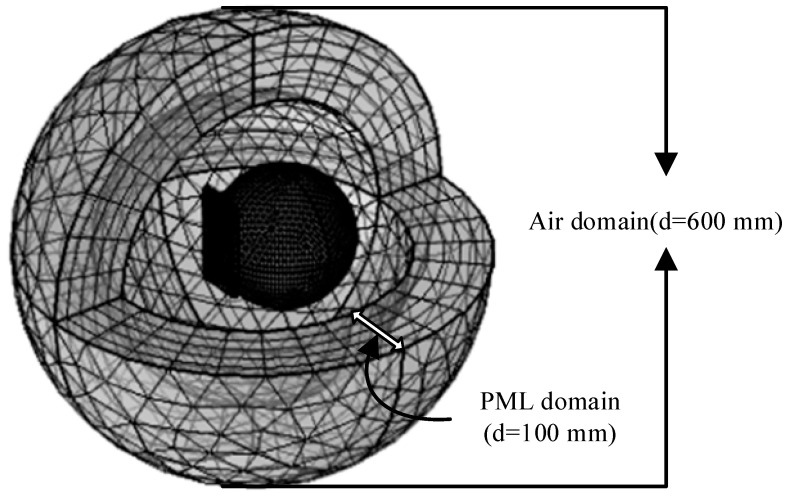
Mesh section of human head model, mobile phone model, and air domain (PML domain).

**Figure 5:**
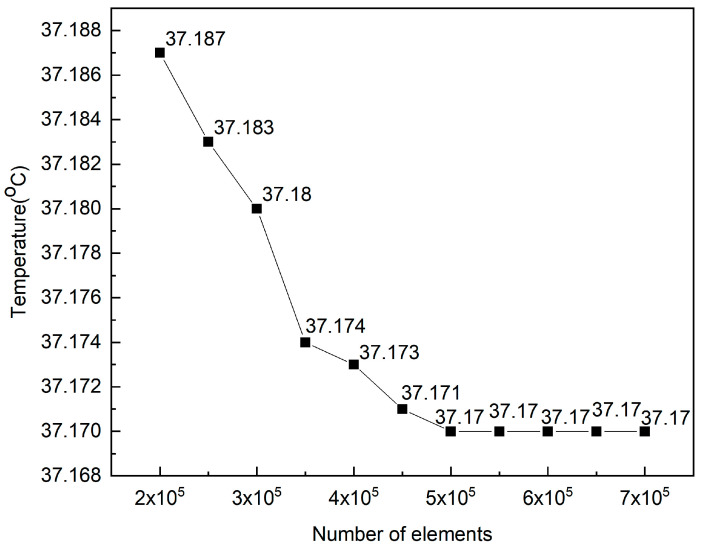
The convergence curve of the model.

This paper uses COMSOL Multiphysics 6.3 for a numerical solution. In this paper, the mobile phone model (including dielectric substrate, antenna, composite silicone substract, glass, ABS shell) and the human head model are modeled in three dimensions in COMSOL Multiphysics 6.3 software through Multiphysics coupling of the RF module. Maxwell’s equations are used to calculate the SAR distribution of the electromagnetic radiation of the 5G mobile phone antenna on each tissue layer of the human head when the input power of the 5G mobile phone antenna is 21 dBm and 24 dBm and the working frequency is 3500 MHz. The head model, mobile phone model, and air domain were meshed using a relatively fine free mesh, as shown in Figure 4. Due to the large computational load, the solution process took more than 30 min on a computer with 16 GB of memory and 4 cores. Convergence tests were also conducted to determine the rationality of the mesh refinement. Considering that the computational load would gradually increase with the increase of the mobile phone antenna input power, the antenna operating frequency and maximum input power in this study were chosen to be 3500 MHz and 24 dBm, respectively. The mesh refinement settings in COMSOL Multiphysics 6.3 were varied from “extremely coarse” to “extremely fine.” The simulation results are shown in Figure 5; Figure 5 shows that when the mesh element count reached 500,000 (i.e., the free mesh refinement was set to relatively fine), the simulation results were independent of the number of mesh elements. Therefore, the mesh refinement accuracy set in this paper is reasonable.

To verify the accuracy of the research method of the constructed model, this paper compared it with the research results published by Bhargavab et al. A rectangular microstrip antenna model identical to that proposed by Bhargavab et al. was established, with the antenna input power, operating frequency, and antenna distance from the human head model set to the same values of 1 W, 900 MHz, and 10 mm, respectively. SAR values for the scalp, skull, and brain layers were obtained through simulation calculations and compared with the SAR values obtained by Bhargavab et al., as shown in Table 3.

As can be seen from the table, the error ratios of the SAR values for the scalp, skull, and brain layers are 1.25%, 0.16%, and 2.12%, respectively. The error ratios show that the obtained results have a high degree of consistency with the published results of Bhargavab et al., thus providing a reliable basis for the research methods and accuracy of the results of the model constructed in this paper.

### 3.2. Mobile Phone Antenna Radiation Characteristics

**Figure 6:**
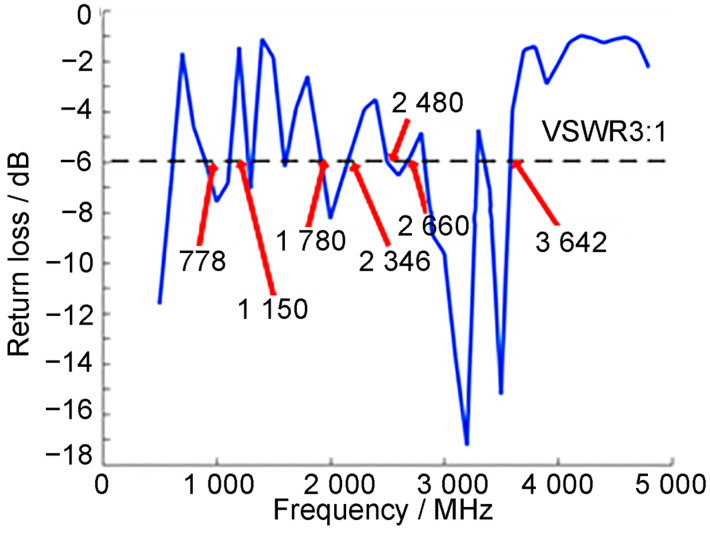
S_11_ resonance curves of 5G mobile phone antenna.

The radiating patch antenna, printed on the top of the phone’s dielectric substrate with dimensions of 52.5 × 15 mm^2^, serves as the near-field radiation source. As illustrated in Figure 6, the resonance curve of the designed antenna shows that with a −6 dB return loss bandwidth (VSWR 3:1) [13], it can cover multiple frequency bands, including GSM-850/900, DCS-1800, PCS-1900, UMTS-2000, WiMAX-2300, LTE-2300/2500, as well as the 3.5 GHz band typically used for 5G mobile communications. Compared with actual 5G mobile phones, the proposed antenna fully satisfies the operational requirements for 5G network frequency bands. We note that both the antenna’s radiation efficiency and the dielectric properties of biological tissues are frequency-dependent. Therefore, while the antenna model is capable of operating across multiple bands, a full multi-band exposure assessment constitutes a significant and necessary extension of this work, which we explicitly recommend for future study.

**Figure 7:**
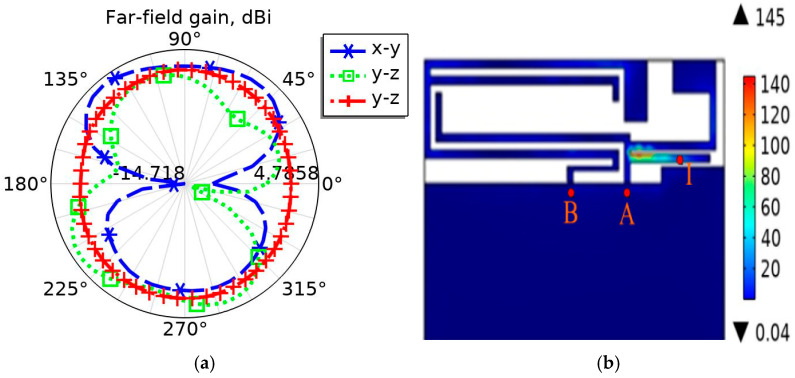
The operating frequency of the 5G mobile phone antenna is 3500 MHz, (**a**) is the radiation pattern in the x-y plane, y-z plane, and x-z plane, (**b**) is the surface current density mode distribution diagram.

Figure 7a shows the radiation pattern of the mobile phone antenna designed in this article in the x-y plane, y-z plane, and x-z plane when the operating frequency is 3500 MHz. It can be seen from the figure that the directional roundness of the antenna is relatively good, and although there are ripples, it is close to omnidirectional. It meets the requirement that the average gain of mobile phone antenna directivity is greater than −3 dBi. Figure 7b shows the surface current density mode distribution diagram when the 5G mobile phone antenna operating frequency is 3500 MHz. It can be seen from the figure that the strong current flows along the path B-A-I, and its length is calculated to be approximately 20 mm, which also corresponds to a quarter wavelength of 3500 MHz. Therefore, according to the radiation pattern of the mobile phone antenna and the current density mode distribution diagram of the antenna surface in Figure 7a,b, the rationality of the antenna design is verified when the antenna operating frequency is 3500 MHz.

### 3.3. SAR Distribution of the Human Head Model

#### 3.3.1. Electric Field Distribution

**Figure 8:**
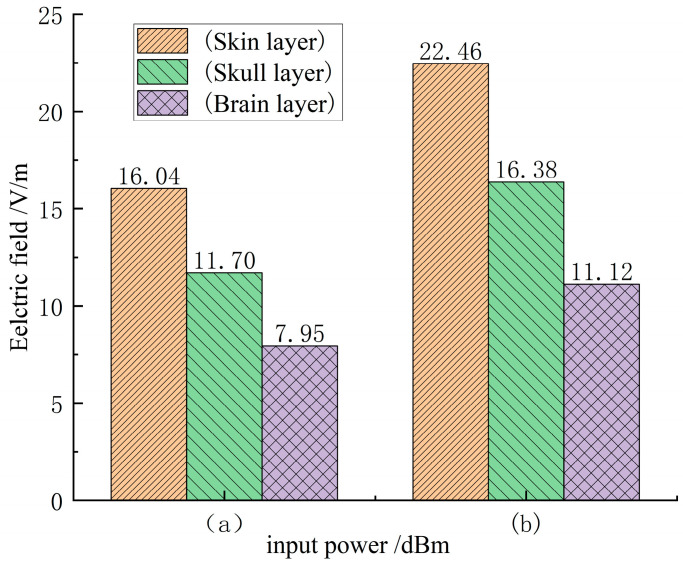
Electric field mode distribution of each tissue layer of the human head model at a distance of 5 mm from the mobile phone antenna and an operating frequency of 3500 MHz, where (**a**) input power is 21 dBm and (**b**) input power is 24 dBm.

In order to perform numerical analysis on the SAR values of each tissue layer of the human head model, it is necessary to calculate the electric field distribution of each tissue layer of the human head Figure 8a,b show the electric field mode distribution of the scalp layer, skull layer, and brain layer when the input power is 21 dBm and 24 dBm, the distance between the mobile phone antenna and the human head is 5 mm, and the antenna operating frequency is 3500 MHz. As shown in the figure, the electric field modes of the corresponding tissue layers differ depending on the antenna input power, and the electric field mode of the scalp layer is always higher than that of the skull layer and the brain layer. When the input power is 24 dBm, compared with the input power of 21 dBm, the electric field modes of each tissue layer at the antenna operating frequency of 3500 MHz are increased by about 0.4 times. In addition, it is noted that as the electromagnetic waves emitted by the mobile phone antenna penetrate the skin layer, enter the skull layer, and finally reach the brain layer, the electric field mode gradually decreases because the reduced electromagnetic energy is absorbed by the tissue layers and converted into resistance heat.

#### 3.3.2. SAR Distribution

**Figure 9:**
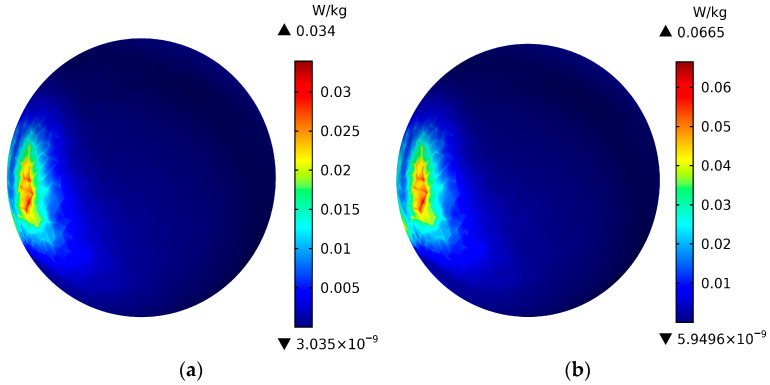
SAR distribution of the brain layer of the human head model at a distance of 5 mm from the antenna and an operating frequency of 3500 MHz. (**a**) represents the antenna input power of 2l dBm, and (**b**) represents the antenna input power of 24 dBm.

The electromagnetic waves emitted by the antenna pass through the scalp layer and skull layer of the human head model and enter the brain layer. As one of the most important parts of the human body, whether the SAR value of the brain exceeds the ICNIRP standard (2 W/Kg) is directly related to whether it will affect human health, Figure 9 shows the SAR distribution of the brain layer of a human head model at a distance of 5 mm from the antenna and an operating frequency of 3500 MHz. (a) represents the antenna input power at 21 dBm, and (b) represents the antenna input power at 24 dBm. As can be seen from Figure 9, when the antenna operating frequency is 3500 MHz, the SAR value is 0.034 W/Kg with an input power of 21 dBm, and 0.065 W/Kg with an input power of 24 dBm. Comparing Figure 9a,b, it can be seen that at a distance of 5 mm from the head and operating at the same frequency, the SAR value of the brain layer with an input power of 24 dBm is approximately twice that with an input power of 21 dBm, Furthermore, it can be seen that regardless of whether the antenna input power is 21 dBm or 24 dBm, the SAR value of the brain layer does not exceed the ICNIRP standard (2 W/Kg).

**Figure 10:**
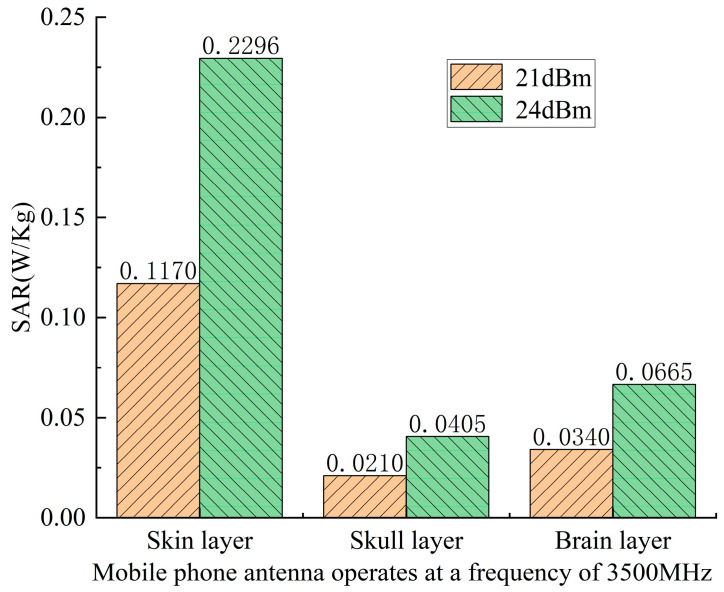
Bar graphs of SAR values of the scalp, skull, and brain layers of the human head model when the mobile phone antenna operates at a frequency of 3500 MHz and the input power is 21 dBm and 24 dBm, respectively.

As shown in Figure 10, as the electromagnetic waves emitted by the mobile phone antenna penetrate the human head model, the SAR value is highest in the scalp layer, decreases rapidly after passing through the skull layer, and increases again after entering the brain layer, but remains lower than the SAR value in the scalp layer, This is determined by the dielectric properties of the skull layer, a large amount of electromagnetic waves are absorbed by the skull layer, thus reducing the amount of electromagnetic waves entering the brain layer, Compared to an antenna input power of 21 dBm, at 24 dBm, the SAR values of all tissue layers at the same operating frequency are greater than those at 21 dBm. This also reflects that as the input power of the mobile phone antenna increases, the SAR value of the mobile phone electromagnetic radiation on the human head gradually increases.

#### 3.3.3. Temperature Field Distribution

When electromagnetic waves emitted by the mobile phone antenna penetrate the human head model, each layer of biological tissue absorbs the electromagnetic radiation energy and converts it into heat loss due to the electromagnetic coupling effect. In order to investigate the temperature field distribution in the skin layer, skull layer, and brain layer of the human head model, this study employs the Pennes transient bio-heat equation (Formula (7)) for numerical simulation. The temperature field distribution in these tissue layers was examined when the 5G mobile phone antenna operated at 3.5 GHz.

**Figure 11:**
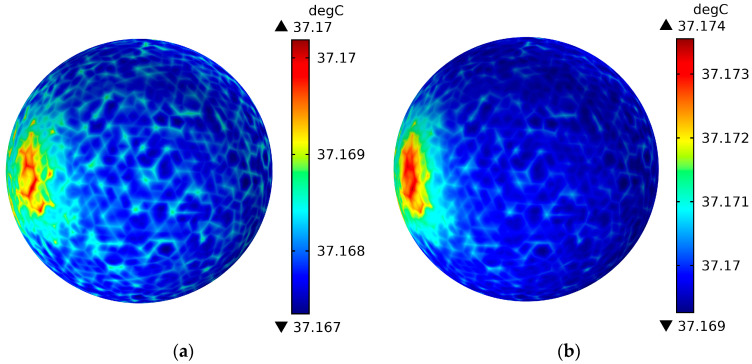
The surface temperature field distribution of the brain layer of the human head model when the mobile phone antenna operates at 3500 MHz and radiates for 30 min. (**a**) is the antenna input power of 21 dBm, and (**b**) is the antenna input power of 24 dBm.

**Figure 12:**
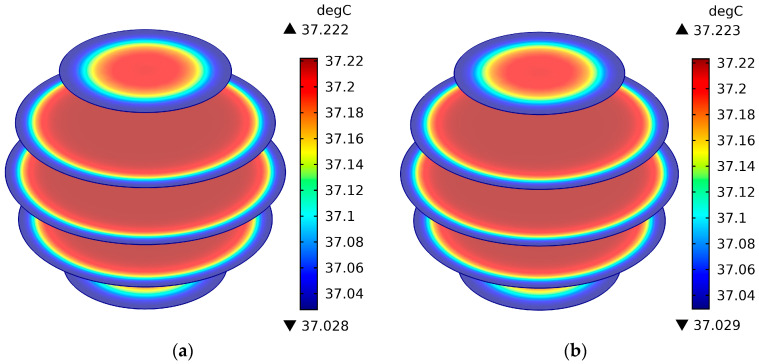
The temperature field distribution of the cross-section of the head model when the mobile phone antenna operates at 3500 MHz and radiates for 30 min. (**a**) is the antenna input power of 21 dBm, and (**b**) is the antenna input power of 24 dBm.

Figure 11 shows the surface temperature field distribution of the brain layer of the human head model when the mobile phone antenna operates at 3500 MHz and radiates for 30 min. (a) is the antenna input power of 21 dBm, and (b) is the antenna input power of 24 dBm. Figure 12 shows the temperature field distribution of the cross-section of the head model when the mobile phone antenna operates at 3500 MHz and radiates for 30 min. (a) is the antenna input power of 21 dBm, and (b) is the antenna input power of 24 dBm. Combining the SAR value distribution of the human head model in Figure 9 and Figure 10, comparative analysis can be seen that the brain layer surface temperature of the brain layer of the human head model is the largest when the antenna operating frequency is 3500 MHz and the antenna input power is 24 dBm. The maximum surface temperature is 37.174 °C, which has a one-to-one correspondence with the corresponding SAR value. At the same time, considering that the heat transfer properties of the tissue in Table 2 have a great impact on temperature, it can be seen from the figure that the brain layer is the most temperature-sensitive area. After 30 min of radiation, the temperature value of the brain layer is higher than that of the scalp cortex and skull layer, and at 3500 MHz, the antenna input power is 24 dBm. The temperature of the brain layer is the highest, reaching 37.223 °C, but it is far lower than the thermal damage temperature of 3.5 °C [5].

**Figure 13:**
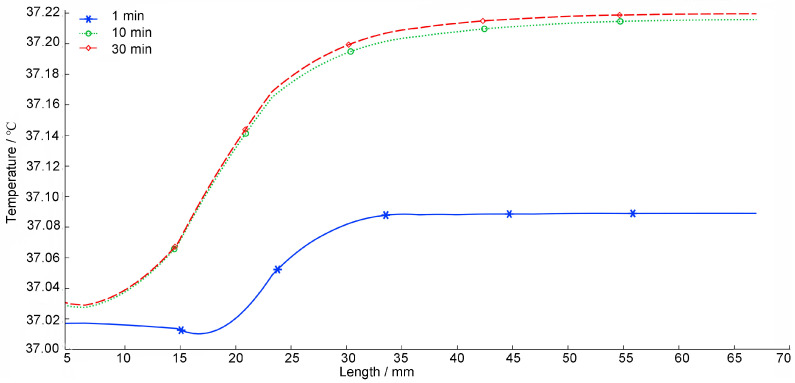
Temperature field change of the horizontal cross-section of the human head model with the radiation time.

Figure 13 studies the temperature field changes in the horizontal cross-section of the human head model as the radiation time changes when the mobile phone antenna operating frequency is 3500 MHz and the antenna input power is 21 dBm. It can be seen from the figure that as the radiation time gradually increases, the biological tissue absorbs electromagnetic radiation energy and the temperature gradually increases. Before the irradiation time was 1 min, the temperature increase of the scalp layer was the highest compared with the skull layer and brain layer. However, when the radiation time exceeds 10 min, the temperature increase of the scalp layer of the human head model is lower than that of the brain layer. This is because the higher the blood irrigation rate of biological tissue, the lower the temperature [7,8,9]. From Table 2, it can be seen that the blood irrigation rate of the scalp is 0.02 1/s, which is higher than the blood irrigation rate of the brain 0.00883 1/s. When irradiated for 30 min, the changes in the temperature field of each tissue layer of the human head model have become smaller and smaller than those at 10 min.

### 3.4. The Impact of Distance on the Head Model

**Figure 14:**
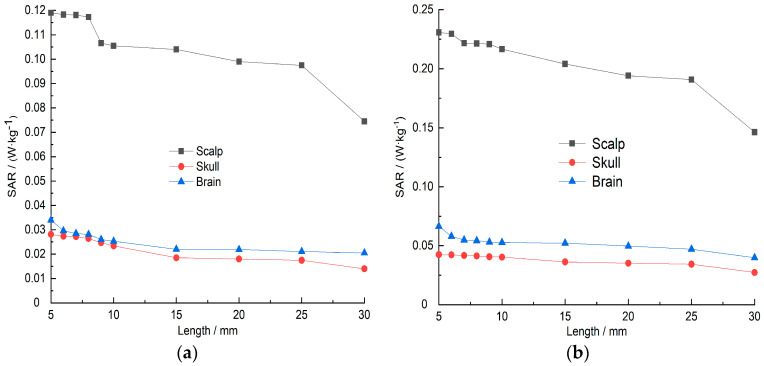
SAR values change of scalp layer, skull layer and brain layer of human head model with distance: (**a**) input power is 21 dBm; (**b**) input power is 24 dBm.

**Figure 15:**
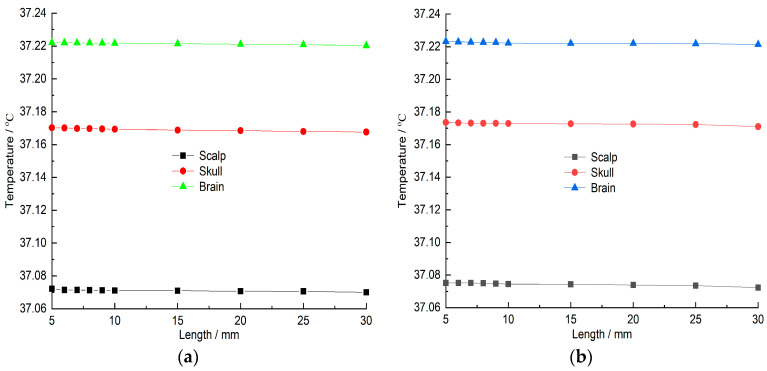
Temperature field change of scalp layer, skull layer and brain layer of human head model with distance: (**a**) input power is 21 dBm; (**b**) input power is 24 dBm.

In the above studies, the 5G hand antenna model was analyzed at a position 5 mm away from the human head model, and the SAR and temperature field distribution of the human head model were analyzed. Figure 14a, Figure 14b, Figure 15a and Figure 15b respectively show the changes in the SAR value and temperature field of the human head model’s scalp cortex, skull layer, and brain layer with the distance from 5 mm to 10 mm (intervals of 1 mm) and 10 mm to 30 mm (intervals of 5 mm) when the mobile phone antenna works in the 5G frequency band (center frequency is 3500 MHz), the antenna input power is 21 dBm and 24 dBm, and the radiation is 30 min. It can be seen from Figure 14 and Figure 15 that as the distance increases, the SAR and temperature of each tissue layer of the human head model show a decreasing trend. It can be seen from Figure 14a,b that when the mobile phone antenna is 5~10 mm away from the human head model, the SAR values of the scalp layer, skull layer, and brain layer decrease relatively slowly. When the distance changes from 10 mm to 30 mm, the SAR value of each layer of the biological tissue of the human head model decreases relatively quickly. However, the overall trend is that as the distance increases, the SAR values of each tissue layer of the human head model decrease. When the antenna input power is 21 dBm, the SAR values of the scalp cortex, skull layer, and cerebral layer are reduced by 50.2%, 35.7%, and 38.71% respectively; when the antenna input power is 24 dBm, the SAR values of the scalp cortex, skull layer, and cerebral layer are reduced by 48.3%, 34.7%, and 38.5% respectively. It can be seen from Figure 15 that compared to the change in SAR value in Figure 14, when the distance between the mobile phone antenna and the human head model increases from 5 mm to 30 mm, the temperature of each tissue layer of the human head model changes very slowly. When the antenna input power is 21 dBm, the temperature fields of the scalp cortex, skull layer, and brain layer only decrease by 0.006%, 0.007%, and 0.18% respectively; when the antenna input power is 24 dBm, the temperature fields of the scalp cortex, skull layer, and brain layer only decrease by 0.004%, 0.005%, and 0.15% respectively.

### 3.5. Limitations and Considerations for Realistic Exposure Assessment

The exposure metrics presented in this work are derived under the assumption of a continuous-wave source, which serves as a standardized and reproducible benchmark. It is important to contextualize these findings within the framework of actual 5G New Radio (NR) transmissions. Commercial 5G systems employ complex modulated waveforms, primarily Orthogonal Frequency-Division Multiplexing (OFDM), characterized by a high Peak-to-Average Power Ratio (PAPR) and a non-constant envelope. This results in significant temporal variations in the instantaneous radiated power. While the time-averaged SAR is expected to correlate with results from a CW analysis of equivalent average power, the transient peak field strengths associated with OFDM symbols could influence localized, short-term energy deposition patterns and related transient thermal responses in tissue. These dynamics represent an additional layer of complexity beyond the scope of the present steady-state model. Therefore, future investigations aiming for the highest fidelity in exposure assessment should incorporate detailed temporal models of specific 5G NR modulation schemes, resource allocation patterns, and traffic bursts to evaluate their potential effects on peak spatial and temporal exposure metrics.

## 4. Conclusions

This study designed a smartphone-integrated antenna model capable of operating across multiple communication bands, including GSM-850/900, DCS-1800, PCS-1900, UMTS-2000, WiMAX-2300, LTE-2300/2500, and the 5G-representative frequency of 3.5 GHz. The specific absorption rate (SAR) distributions in the scalp, skull, and brain layers of a human head model were systematically evaluated under near-field exposure at 3500 MHz with input powers of 21 dBm and 24 dBm, at a separation distance of 5 mm. The results indicate that the SAR in the brain layer at 24 dBm was approximately twice that at 21 dBm. In both cases, the highest SAR occurred in the scalp layer, yet all values remained well below the ICNIRP safety limit of 2 W/kg. Moreover, the maximum SAR observed in the brain layer corresponded to the peak surface temperature in the same region; however, this temperature increase remained within a physiologically negligible range and poses no risk of thermal injury.

Further analysis revealed that after 10 min of exposure, the temperature rise in the scalp layer became lower than that in the brain layer. Following 30 min of radiation, the temperature variation across the head model progressively stabilized, showing only minimal changes compared to the 10-min mark. In terms of distance dependence, when the antenna–head distance increased from 5 mm to 10 mm (in 1 mm steps), the decrease in SAR across all tissue layers was relatively gradual. A more pronounced reduction up to 50.2% was observed when the distance was extended from 10 mm to 30 mm (in 5 mm steps). In contrast, the corresponding temperature changes across tissues were markedly less sensitive to distance, with a maximum decrease of only 0.18% over the same range.

In conclusion, this study employs a canonical multi-layer spherical head model to perform a systematic, parametric analysis of electromagnetic exposure from a 5G mobile phone antenna at 3.5 GHz. The results provide clear quantitative trends regarding the dependence of SAR and temperature rise on key variables such as input power and antenna-to-head distance. It is important to interpret these findings as illustrative of fundamental biophysical interactions under controlled conditions, rather While the simplified geometry of our head model dictates that the absolute SAR and temperature values are scenario-specific, the key relationships and patterns we identify are robust and physically meaningful. These patterns offer crucial insights for safety-by-design principles in antenna development and help contextualize the rationale behind standardized compliance testing. Moving forward, extending this parametric approach to diverse anatomical models and higher 5G frequency bands will be essential for building a comprehensive, evidence-based understanding of human exposure in next-generation wireless networks.

Furthermore, the evolution toward advanced antenna systems, such as those employing beamforming and massive MIMO, opens a promising frontier for proactive exposure control. in the future, our work will explore the paradigm of “Exposure-Aware Beamforming.” This would involve developing intelligent algorithms that leverage real-time sensor or channel data to infer device-body orientation and dynamically steer beams away from the user, thereby integrating safety as an active optimization goal within the communication system itself.

## Figures and Tables

**Table 1:** Dielectric properties of human tissue layers at 3500 MHz.

Frequency	Tissue	Relative Permittivity	Conductivity (S/m)
3500 MHz	skin	37.005	2.025
	skull	17.43	1.199
	brain	41.154	2.223

**Table 2:** Thermal properties of different biological tissues of the human head model [7].

Tissues	$\rho \;{(\mathrm{kg}/m}^{3})$	k (w/m·°C)	C (J/kg·°C)	$Q_{met}\;{(w/m}^{3})$	$\omega_{b}\;(1/s)$
Scalp	1125	0.42	3600	1620	0.02
Skull	1990	0.37	3100	610	0.000463
Brain	41,038	0.53	3650	7100	0.00883

**Table 3:** Comparison of calculated SAR values with those of Bhargavab et al. [7].

Tissue	Bhargavab	This Paper	Error Ratio
Scalp	1.58 (W/kg)	1.60 (W/kg)	1.25%
Skull	0.17 (W/kg)	0.16 (W/kg)	0.16%
Brain	0.46 (W/kg)	0.47 (W/kg)	2.12%

## Data Availability

The original contributions presented in this study are included in the article. Further inquiries can be directed to the corresponding author.
